# Hsa_circ_0000497 and hsa_circ_0000918 contributed to peritoneal metastasis of ovarian cancer via ascites

**DOI:** 10.1186/s12967-022-03404-9

**Published:** 2022-05-10

**Authors:** Ning Luo, Zubaidan Sulaiman, Chunyan Wang, Jinye Ding, Yingying Chen, Biting Liu, Zhongping Cheng, Shupeng Liu

**Affiliations:** 1 Department of Obstetrics and Gynecology, Shanghai Tenth People’s Hospital, School of Medicine, Tongji University, Shanghai, 200072 China; 2 Institute of Gynecological Minimally Invasive Medicine, School of Medicine, Tongji University, Shanghai, 200072 China; 3 Department of Obstetrics and Gynecology, Putuo People’s Hospital, Tongji University, Shanghai, 200060 China

**Keywords:** Ovarian cancer, Circular RNA, Epithelial-Mesenchymal Transition, Peritoneal metastasis, Ascites

## Abstract

**Purpose:**

As a common complication of epithelial ovarian cancer (EOC), malignant ascites contributes to the peritoneal metastasis of EOC. CircRNAs play essential roles in tumor metastasis. However, no circRNAs have been reported to be involved in EOC peritoneal metastasis via ascites.

**Methods:**

Total of 22 samples from 9 EOC patients containing primary lesions (T), tumor cells from ascites (ASC), and metastatic lesions (M) were included for RNA sequencing to identify differentially expressed circRNAs and mRNAs among different tumors. Bioinformatic analyses, including single-sample Gene Set Enrichment Analysis and soft cluster analysis, were performed to find circRNAs potentially correlated with ascitic metastasis. Wound healing and transwell analysis were performed to evaluate tumor cells metastasis in vitro. Quantitative real-time PCR and western-blot were used for gene expression evaluation.

**Results:**

According to transcriptomic analysis, ASC showed mesenchymal phenotype while T and M showed epithelial phenotype. 10 circRNAs were differentially expressed among ASC, T, and M. Among them, hsa_circ_0000497 and hsa_circ_0000918 were significantly up-regulated in ASC. Functional analysis showed that both hsa_circ_0000497 and hsa_circ_0000918 promoted metastasis of EOC via epithelial-mesenchymal transition (EMT) in vitro. The regulatory network construction identified 8 miRNAs and 19 mRNAs, and 7 miRNAs and 17 mRNAs as potential downstream target genes of hsa_circ_0000497 and hsa_circ_0000918, respectively, which may play pivotal roles in EOC ascitic metastasis.

**Conclusions:**

circRNAs (hsa_circ_0000497 and hsa_circ_0000918) contribute to metastasis of EOC via ascites by regulating EMT. These circRNAs may serve as novel potential therapeutic targets or prognostic biomarkers for EOC peritoneal metastasis.

**Supplementary Information:**

The online version contains supplementary material available at 10.1186/s12967-022-03404-9.

## Introduction

Epithelial ovarian cancer (EOC) is the most lethal gynecological malignancy. Five years survival of EOC is only 29% due to its high invasive and metastatic feature [1]. Approximately 75% of EOC patients were diagnosed with peritoneal metastasis, which was correlated with a poor prognosis [2]. Pelvic-peritoneal cavity transcoelomic route is regarded as the typical way of EOC metastasis, which is widely believed to occur via peritoneal circulation of ascites [3]. Malignant ascites was a common complication of EOC and was reported to be an independent unfavorable prognostic factor of EOC [4, 5]. Constant Malignant ascites led to metastasis [6–8], while the underlying mechanism needs more thorough investigation.

Ascites contains tumorigenic cytokines, proteinases, chemokines, and growth factors and function as a metastatic tumor microenvironment [9], which provides eligible conditions for tumor expansion, migration, and cell proliferation [10]. Ascites has been shown to promote EOC metastasis by reducing junctional proteins, including connexin 43 (gap junction), E-cadherin (adherent junction), occludin (tight junction), and desmoglein (desmosome) [11]. EOC cells detached from the primary site to ascites via Epithelial-Mesenchymal Transition (EMT), translocated to peritoneum via ascites circulation and formed metastasis via Mesenchymal- Epithelial Transition (MET) [12]. Spheroids isolated from ascites of EOC patients have confirmed EMT features [13]. VEGF in EOC patients was positively correlated with the development of malignant ascites and increased peritoneal permeability through the down-regulating of claudin 5 and E-cadherin in the peritoneal endothelium [14]. Ascites was also reported to induce stem-cell-like phenotype of tumor cells via EMT [15]. These reports evidenced that ascites was involved in promoting metastasis via regulating EMT.

Circular RNA (circRNA), a novel class of endogenous RNAs, was found to be involved in multiple biological processes, including tumor development and metastasis. Increasing evidence showed that circRNAs promoted cancer cell metastasis by regulating EMT [16, 17]. Transcription factors such as Snail [18] and vimentin [19] and pivotal signaling pathways, including TGF-β/Smad pathway [20] and AKT1/mTOR pathway [21] involved in EMT, were found to be regulated by circRNAs in tumor cells. CircCul2, up-regulated by Twist, was reported to increase the expression of vimentin to promote EMT in hepatocellular carcinoma [19]. circPTK2 promoted EMT of colorectal cancer cells by up-regulating vimentin expression via physically binding to vimentin [16]. And several circRNAs such as hsa_circ_0061140 [22], circFGFR3 [23], hsa_circ_100395 [24], hsa_circ_0025033 [25], and hsa_circ-0005585 [26] were all reported to promote ovarian cancer metastasis via EMT. These results indicated that up-regulated circRNAs in tumor tissues had pivotal roles in promoting metastasis of tumors including EOC. However, circRNAs expressed in tumor cells from ascites and involved in peritoneal metastasis of EOC via ascites remain unidentified.

In the present study, we found two circRNAs, hsa_circ_0000497 and hsa_circ_0000918, significantly up-regulated in ascites tumor cells from EOC patients via transcriptomic analysis. Expression of hsa_circ_0000497 and hsa_circ_0000918 were up-regulated in ascites tumor cells compared with primary and metastatic tissues. The overexpression of these circRNAs promoted tumor cell metastasis via EMT in vitro. In addition, we constructed a regulatory network including miRNAs and mRNAs for each circRNA and identified the potential downstream targets of these circRNAs, which may contribute to the promotional role of circRNAs in tumor cell metastasis. These findings indicated that hsa_circ_0000497 and hsa_circ_0000918 contribute to peritoneal metastasis of EOC via ascites by regulating EMT of tumor cells, which can be used as novel therapeutic targets or prognostic biomarkers of EOC peritoneal metastasis.

## Materials and methods

### Patients and data collection

**Fig. 1:**
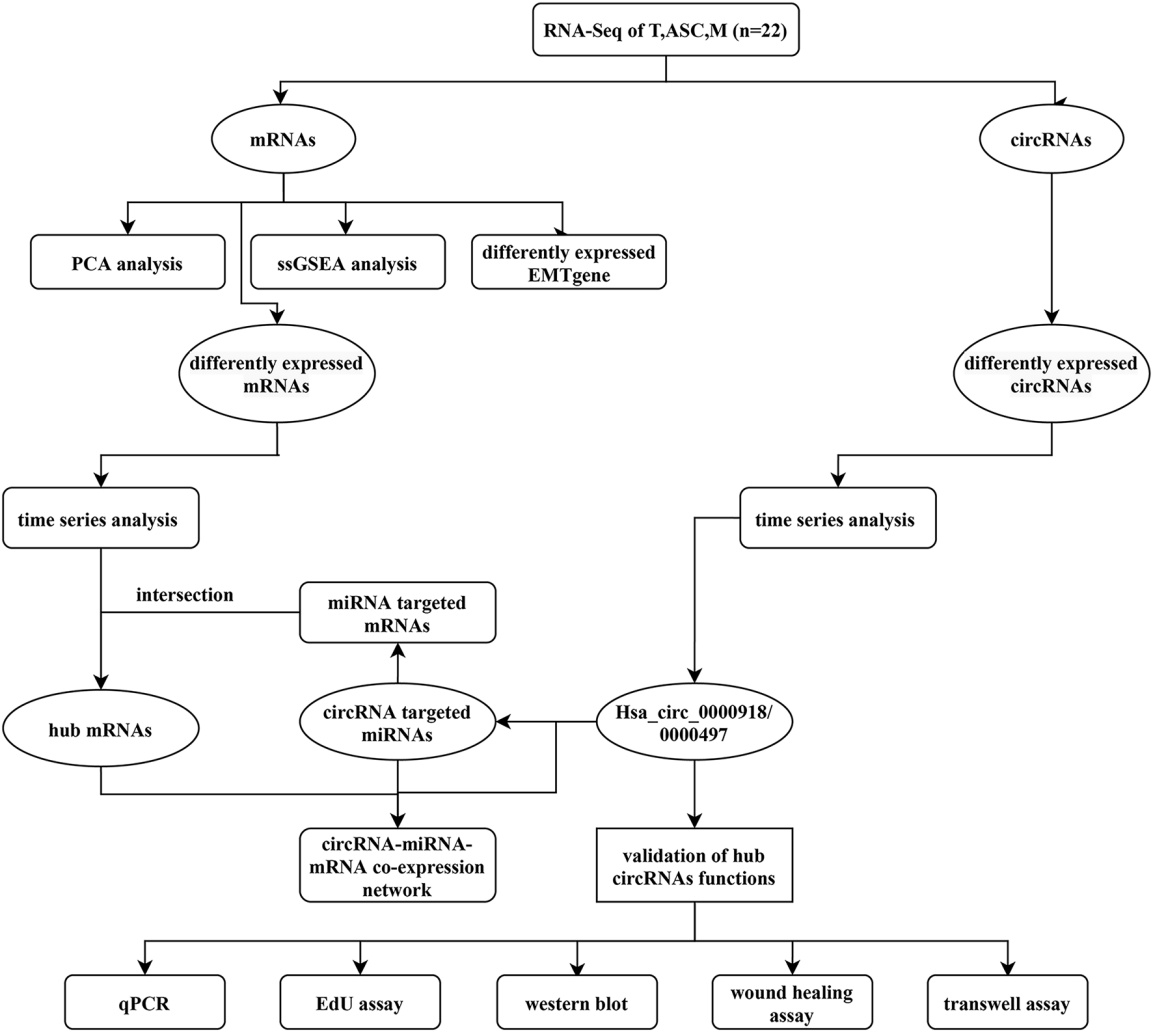
workflow of this study

4 sets of paired samples, including primary tumor lesions (T), ascites tumor cells (ASC), and metastatic lesions (M), and an additional 5 pairs of T and M were obtained from 9 EOC patients for RNA-sequencing. And 8 sets of paired samples, including T, ASC and M were included for validation. All EOC patients were informed at the Department of Gynecology and Obstetrics of Tenth People`s Hospital of Shanghai. And the histopathological diagnosis of EOC was based on the World Health Organization criteria [27].This study was approved by the Ethics Committee of Shanghai Tenth People’s Hospital. The pathological classification of T and M was confirmed by veteran pathologists. Ascites was obtained during surgery and centrifuged at 3000 rpm for 5 min (mins), then the supernatant was carefully removed, and precipitant (cells in ascitic fluid) left for research. The detailed clinical information of patients was listed in Table 1. The overall analysis workflow was shown in Fig. 1.

**Table1:** The detailed clinical information for patients

Patient_id	Sample_id	Age	Stage	Diagnosis
P1	T1, ASC1, M1	57	IIIc	HGSOC
P2	T2, ASC2, M2	64	IIIc	HGSOC
P3	T3, ASC3, M3	71	IIIc	HGSOC
P4	T4, ASC4, M4	78	IIIc	HGSOC
P5	T5, M5	67	IIIc	HGSOC
P6	T6, M6	62	Ia	HGSOC
P7	T7, M7	62	IIIc	HGSOC
P8	T8, M8	77	IIIc	HGSOC
P9	T9, M9	63	III	HGSOC
P10	T, ASC, M	63	IV	HGSOC
P11	T, ASC, M	55	IV	HGSOC
P12	T, ASC, M	64	IIIc	HGSOC
P13	T, ASC, M	48	IIIa	HGSOC
P14	T, ASC, M	59	IV	HGSOC
P15	T, ASC, M	77	IV	HGSOC
P16	T, ASC, M	77	IIIc	HGSOC
P17	T, ASC, M	52	IIIc	HGSOC

### Cell lines and cell culture

EOC cell lines SKOV3 and OVCAR3 were cultured under a 37 °C with 5% CO_2_ and at least 95% humidity in RPMI1640 (Gibco, USA) supplemented with 10% FBS (BI, China). Both cell lines were qualified by STR (HuaKe Gene technology, China) before further analysis.

### Total RNA extraction and quantitative real-time PCR

Total RNA of samples was extracted using RNAiso Plus (TaKaRa, Japan) according to the manufacturer's instructions. The concentration and purity of all RNA samples were subsequently measured by NanoDrop2000 (Thermo Scientific, USA). 1 ug of total RNA was used for cDNA (Complementary DNA) synthesis using PrimeScript RTMaster Mix (TaKaRa, Japan). Quantitative real-time PCR (qRT-PCR) was performed on a QuantStudio Dx (ABI, America) using SYBR Premix ExTaq kit (Takara, Japan). The relative expression was calculated by the 2^−△△Ct^ method with GAPDH reference control. All primer sequences were listed in Additional file 1: Table S1.

### Western blot assay

The protein extraction was completed with RIPA buffer (GenePharma, China) with 1% General Protease Inhibitor Cocktail (MKBio, China) on ice and centrifuged at 4 °C, 12,000 rpm/15 min. The concentration of the supernatant was evaluated using the BCA Protein assay kit (Beyotime, China), and then the extracted proteins with 6 × SDS loading buffer (Beyotime, China) were denatured by heating (95 °C) for 10 min. The sample was transferred to the PVDF membrane after SDS-PAGE electrophoresis, blocked with 5% skim milk solution for 100 min, and incubated with primary antibodies at 4 °C overnight. Then, the membranes were incubated with the secondary antibody at room temperature for 1 h. Immobilon ECL substrate (EpiZyme, China) and Amersham Imager 600 (Cytiva, America) were used for signals detection and image acquisition. The antibodies used in this study were listed in Additional file 1: Table S2.

### Plasmid construction and transfection

The pLCDH-ciR (Geneseed, China) was used to create hsa_circ_0000497 and hsa_circ_0000918 overexpressing plasmids. Small interference RNAs (siRNAs) targeting the junction sequence of hsa_circ_0000497 or hsa_circ_0000918 and control siRNA (Additional file 1: Table S1) were designed and synthesized by GenePharma (China). SKOV3 and OVCAR3 were transfected with Lipofectamine 3000 reagent (Invitrogen, USA) according to the manufacturer's instructions.

### Wound healing assay

SKOV3 cells were seeded in 6-well plates with 1 × 10^5^ cells per well. 24 h after transfection, micropipette tips were used to scratch the center to create a straight wound. The cells were cultured in RPMI1640 containing 1% FBS. Cell migration was captured with a microscope imaging system at different time points (0 h and 24 h), and the relative migration rate was calculated according to the formula below [28].

$${\text{Wound closure }}\% = \left( {\frac{{A_{{t = 0 - A_{t = \Delta t} }} }}{{A_{t = 0} }}} \right) \times 100\%$$

Where ${A}_{t=0}$ is the initial wound area, and ${A}_{t=\Delta t}$ is the wound area after 24 h of the initial scratch, both in μm^2^.

### Transwell migration and invasion assays

Cell invasion and migration assays were performed using 24-well transwell plates (Corning, USA) coated with or without Matrigel™ Matrix (Corning, USA). 24 h after transfection, tumor cells (1 × 105) for invasion assay and (5 × 104) for migration assay were trypsinized and washed twice with PBS, suspended in 200 μL serum-free RPMI1640, slowly dripped into the pre-coated insert, and incubated in a 24-well plate containing 600 μL RPMI1640 with 20% FBS per well for 48 h. The matrigel and cells on the upper surface of the membrane were then wiped off. The invasive cells that migrated through the membrane and adhered to the lower surface, and were fixed in 4% paraformaldehyde for 18 min and stained with DAPI (Merck, China). The number of invasive cells was photographed and counted using an inverted microscope imaging quantification field system (magnification: 100, Nikon, Japan).

### EdU assay

The cell proliferation was detected using BeyoClickTM EdU Cell Proliferation Kit with Alexa Fluor 555 (Beyotime, China). SKOV3 cells were seeded in 24-well plates with 7 × 10^4^ cells per well. 48 h after transfection, EdU was applied at 20 μM. The cells were then fixed with 4% paraformaldehyde and stained with Alexa Fluor 555 and DAPI. The cell proliferation was photographed and counted using an inverted microscope imaging quantification field system (magnification: 100; Nikon, Japan).

### RNA-sequencing assay

The next-generation RNA-sequencing was performed in (JRDUN, China). Briefly, total RNA was isolated with the RNAiso Plus (TaKaRa, Japan). The quality was checked using Agilent Bioanalyzer (Thermo, USA). RNA integrity number (RIN) for all samples was more than 7. The Illumina TruSeq Stranded RNA Sample Preparation kit (Illumina, USA) for library preparation. Then the libraries were sequenced using Illumina NovaSeq 6000 (Illumina, USA) with paired-end ($2\times 150$) nucleotide reads. Low-quality reads and residual adapter sequences from FASTQ files were filtered and trimmed using Skewer with default parameters. Read count per RNA was computed using HTSeq [29]. To avoid variation caused by total reads sequenced, raw read counts were normalized to the total read count by sample. Log_2_ transformations were performed on normalized read counts. To avoid the log of zeroes, all read counts were increased by 1 before taking the log transformation. The circRNAs were detected and identified by the *find_circ* algorithm with default parameters [30]. CIRCexplorer2 software was used to predict circular RNAs' front and back positions based on the previous results. Gene annotation for circular RNA is sorted by exon > exon_intron > intron > intergenic region to ensure that the predicted circular RNA is consistent with the strand direction of the known or newly predicted transcripts in the database.

The principal component analysis (PCA) was utilized to emphasize variation and similarity among the 22 samples using R packages *FactoMineR* after the data normalized.

Differentially expressed circRNA (DECs) and differentially expressed genes (DEGs) were identified using R packages *DESeq2* [31] with foldchange > 2 and p value < 0.05. DEGs and DECs volcano plots and heatmaps were visualized using the *grepel, ggplot, and pheatmap* packages. The venn diagram was employed to take the intersection of DECs and DEGs.

### Single sample gene set enrichment (ssGSEA) analysis

Single sample gene set enrichment analysis (ssGSEA) was applied to evaluate the enrichment score of EMT gene signature in each sample. Signatures of epithelial (n = 31) and mesenchymal (n = 54), used for ssGSEA, were connected from previous literature and GO databases (http://geneontology.org/) (Additional file 1: Table S3) [32]. Each geneset's normalized enrichment scores were calculated using R package *GSVA* [33].

### Mfuzz soft clustering analysis

The *Mfuzz* package in R software (http://www.bioconductor.org/packages/release/bioc/html/Mfuzz.html, version 2.50.0) was applied to perform soft clustering analysis of circRNA and mRNA from T, ASC, M expression matrices [34]. RNAs were eliminated if the standard deviation (SD) was < 0.4 and Cluster cores = 0.5. The Fuzzy C‑Means clustering method was adopted to conduct clustering analysis based on time variation and the change in expression levels. Finally, multiple clustering results were obtained, and those with consistent expression changes were placed in the same group. DEGs and DECs were identified according to the difference in expression value (p < 0.05 was used as the cut‑off value).

### Prediction of miRNA binding sites and target genes

Cancer-Specific CircRNAs Database (CSCD) is utilized for visualizing the structure of circRNAs. The circBase (http://www.miranda.org/) was used to identify binding miRNAs of the circRNA candidates. Interactions between miRNA (microRNA) and mRNA were analyzed based on the TargetScan (http://www.targetscan.org) and miRDB databases (miRNA target prediction database) (http://mirdb.org). DEGs recognized in both databases were considered as candidate mRNAs of circRNA-miRNA regulation elements. The circRNA–miRNA–mRNA regulatory network was constructed through combination analysis of circRNA–miRNA pairs and miRNA–mRNA pairs and visualized using Cytoscape 3.8.2 [35].

### Statistical analysis

All experiments were repeated at least three times in this study. Data were shown as mean ± standard error. All statistical analyses were performed using R software (v 0.4.0.3). p < 0.05 was considered to be statistically significant. * indicates p < 0.05, ** indicates p < 0.01, *** indicates p < 0.001 and **** indicates p < 0.0001.

## Results

### Transcriptomics profiling identified EMT-MET in EOC via the peritoneal circulation

**Fig. 2:**
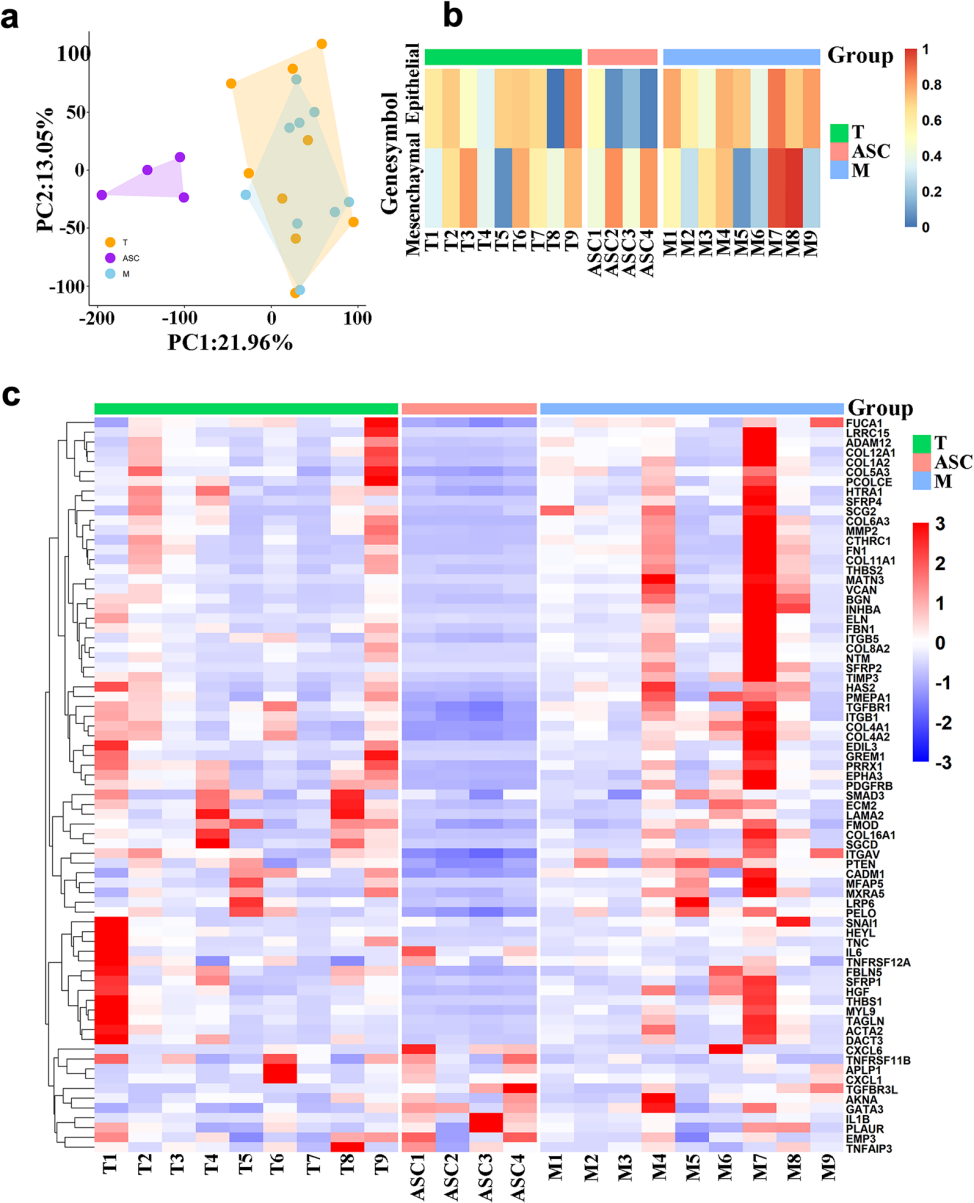
Transcriptomics profiling identifies biological processes of EMT in ovarian cancer via the peritoneal circulation. **a** PCA score plot of the mRNA expression data of primary tumor lesions (T), ascites tumor cells (ASC) and metastatic lesions (M), The convex whole of the sample distributions in PCA. PCA: principal component analysis. Each point represents a dimensional reduction spectrum. All data were separated by sample type, and the automatic combination can better reflect the nature of different tissue cells. **b** Heatmap showing the enrichment levels (ssGSEA scores) of epithelial signatures and mesenchymal signature in mRNA expression of T, ASC, and M. ssGSEA: single sample gene-set enrichment analysis. **c** Heatmap for expression of genes from the EMT signature in T, ASC, and M. Red represents high expression levels; Green represents low expression levels; Each row represents a circRNA; Each column represents the expression profile of a sample

To investigate the dynamic states of epithelial-mesenchymal transition (EMT) during epithelial ovarian cancer (EOC) ascitic metastasis, we performed a comprehensive analysis of gene expression profiles of 22 samples, including 9 primary tumor lesions (T), 4 ascites (ASC), and 9 metastatic lesions (M) from 9 EOC patients. Principal component analysis (PCA) was performed to analyze the pattern of mRNA expression profile. Our results showed that ASC metastasis was distinguished remarkably from T and M, while T basically overlapped with M, indicating that the common essence showed between T and M as solid tumor tissue (Fig. 2a). Increasing evidence showed that EOC gains aggressiveness by losing the epithelial characteristics and acquiring the mesenchymal phenotype [36]. To verify the EMT phase of the samples with ascitic metastasis of ovarian cancer, representative gene sets of the epithelial (E phenotype, gene counts n = 31) and the mesenchymal (M phenotype, gene counts n = 54) were manually collected from previous literature and GO (gene oncology) databases (http://geneontology.org/) to conduct ssGSEA analysis [32]. In accordance with PCA, the enrichment of epithelial phenotype in ascites was significantly lower than that in T and M (Fig. 2a). Meanwhile, the enrichment of mesenchymal phenotype in ASC was considerably higher than that in M (Fig. 2b), while no significant difference was found in the enrichment of epithelial and mesenchymal phenotype gene sets in T (Fig. 2b). Heatmap of EMT-related genes showed a distinct pattern of ASC, which characterized as significant down-regulation of epithelial genes, but up-regulation of mesenchymal genes, compared with T and M, while T and M shared a similar expression pattern of EMT-genes (Fig. 2c). These results indicated that ovarian cancer cells might undergo EMT-MET while migrating to ascites and form distant metastasis via EMT.

### Screening for circRNAs differentially expressed in ascitic metastasis of ovarian cancer

**Fig. 3:**
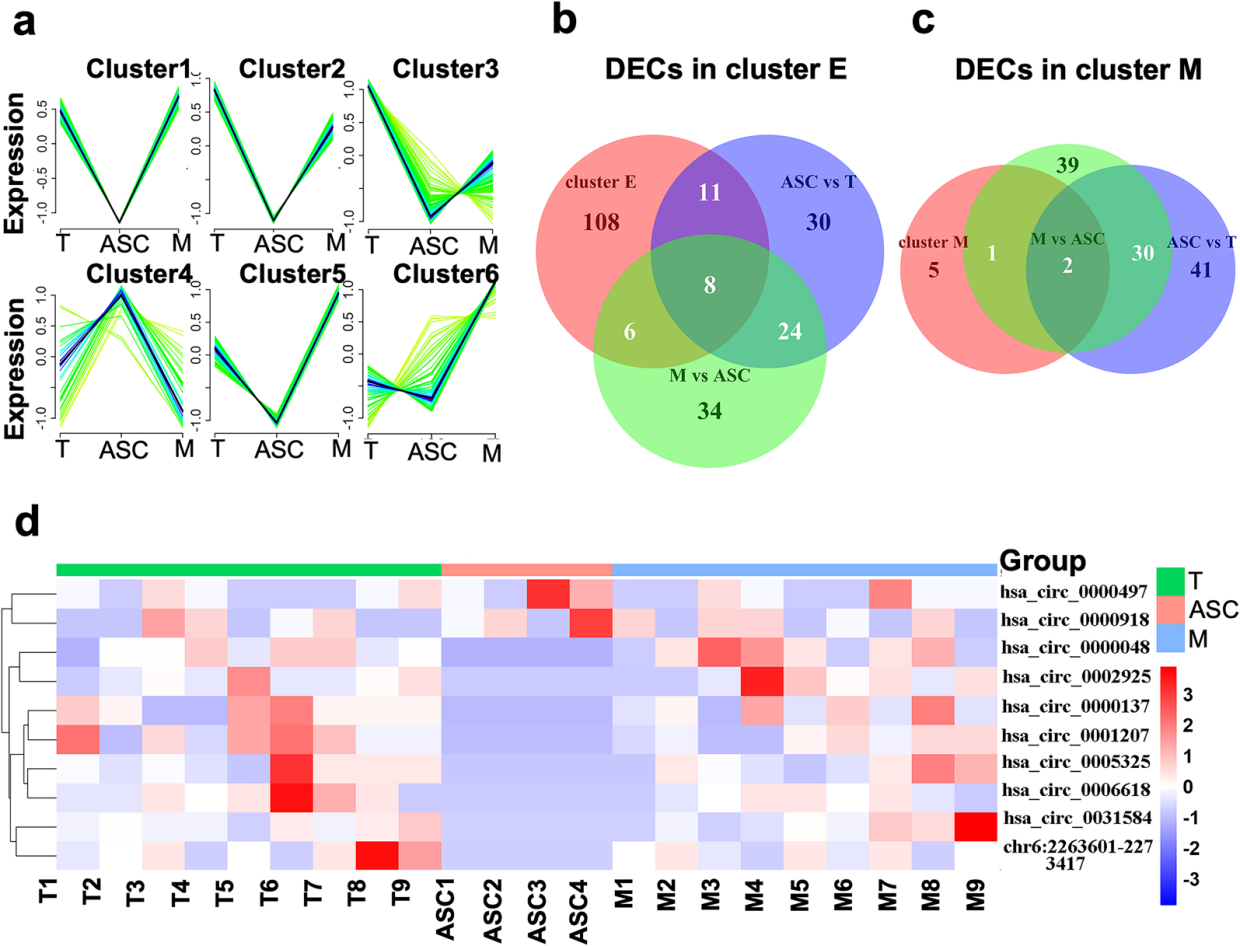
Screening for circRNAs differentially expressed in ASC. **a** Clusters obtained by time clustering analysis for circRNAs expression, Horizontal axis represents samples (T, ASC, and M). The vertical axis represents expression changes. Cluster E (epithelial phenotype, cluster1,2, and 5) and cluster M (mesenchymal phenotype, cluster4), respectively. **b** Venn diagram of overlapped circRNAs among the differentially expressed circRNAs between ASC and T groups, ASC and M groups, and cluster_E groups. **c** Venn diagram of overlapped circRNAs among the differentially expressed circRNAs between ASC and T groups, ASC and M groups, and cluster_M groups. **d** Heatmap for differently expressed circRNAs. Red represents high expression levels; green represents low expression levels; each row represents a circRNA; each column represents the expression profile of a tissue sample

circRNAs have been reported to play an essential role in cancer metastasis [37]. To find out circRNAs contribute to ascites metastasis of ovarian cancer, differentially expressed circRNA (DECs) among the three groups (T, ASC, and M) were determined using *DESeq2* software package. 72 circRNAs (including 5 up-regulated and 67 down-regulated) and 71 circRNAs (including 57 up-regulated and 14 down-regulated) were found differentially expressed in T-ASC group and ASC-M group, respectively (Additional file 1: Fig. S1a, b). Then, soft clustering analysis was carried out using R package *Mfuzz* to identify dynamic circRNAs expression trends in the EOC metastatic progression in T, ASC, and M [38]. According to the tumor proceeding and expression level, the following Cluster analyses identified 6 clusters of circRNAs with a similar trend but a slight difference in expression changes (Fig. 3a). In clusters 1, 2, and 5, circRNAs were highly expressed in T and M groups and lowly expressed in ASC group. In cluster 4, circRNAs were lowly expressed in T and M groups and highly expressed in ASC group (Fig. 3a). Due to the unique expression pattern in T, M, and ASC with different EMT phenotypes, clusters 1, 2, and 5 were named as the epithelial hypomorphic cluster (cluster E), while cluster 4 was named as the mesenchymal hypomorphic cluster (cluster M). Among these circRNAs, a total of 10 circRNAs, including 8 circRNAs from cluster E and 2 circRNAs from cluster M were found to be DECs identified previously (Fig. 3b, c, Table 2). The putative pattern of these circRNAs was further validated using the transcriptomic data (Fig. 3d). The 2 circRNAs, hsa_circ_0000497 and hsa_circ_0000918, were identified as ASC-specific circRNAs with a correlation of EMT score.

**Table 2:** Basic information of candidate circRNAs

CircRNA_Id	Position	Strand	Spliced length	Best transcript	Gene symbol
hsa_circ_0031584	chr14:32559707–32586493	+	4033	NM_001030055	ARHGAP5
hsa_circ_0002925	chr9:115336336–115337531	+	1195	NM_133465	KIAA1958
hsa_circ_0005325	chr19:34921480–34925873	+	321	NM_005499	UBA2
hsa_circ_0000048	chr1:33413822–33415375	−	351	NM_153341	RNF19B
hsa_circ_0000137	chr1:155408117–155408859	−	742	NM_018489	ASH1L
hsa_circ_0081207	chr7:97820925–97823884	+	2959	NM_014916	LMTK2
hsa_circ_0000497	chr13:78293666–78327493	+	788	NM_001040153	SLAIN1
hsa_circ_0000918	chr19:19750893–19751380	−	299	NM_016573	GMIP
hsa_circ_0006618	chr4:83799882–83803093	−	406	NM_001077207	GMDS-AS1
hsa-GMDS-AS1_0002	chr6:2263601–2273417	+	666(estimated)		GMDS-AS1

**Fig. 4:**
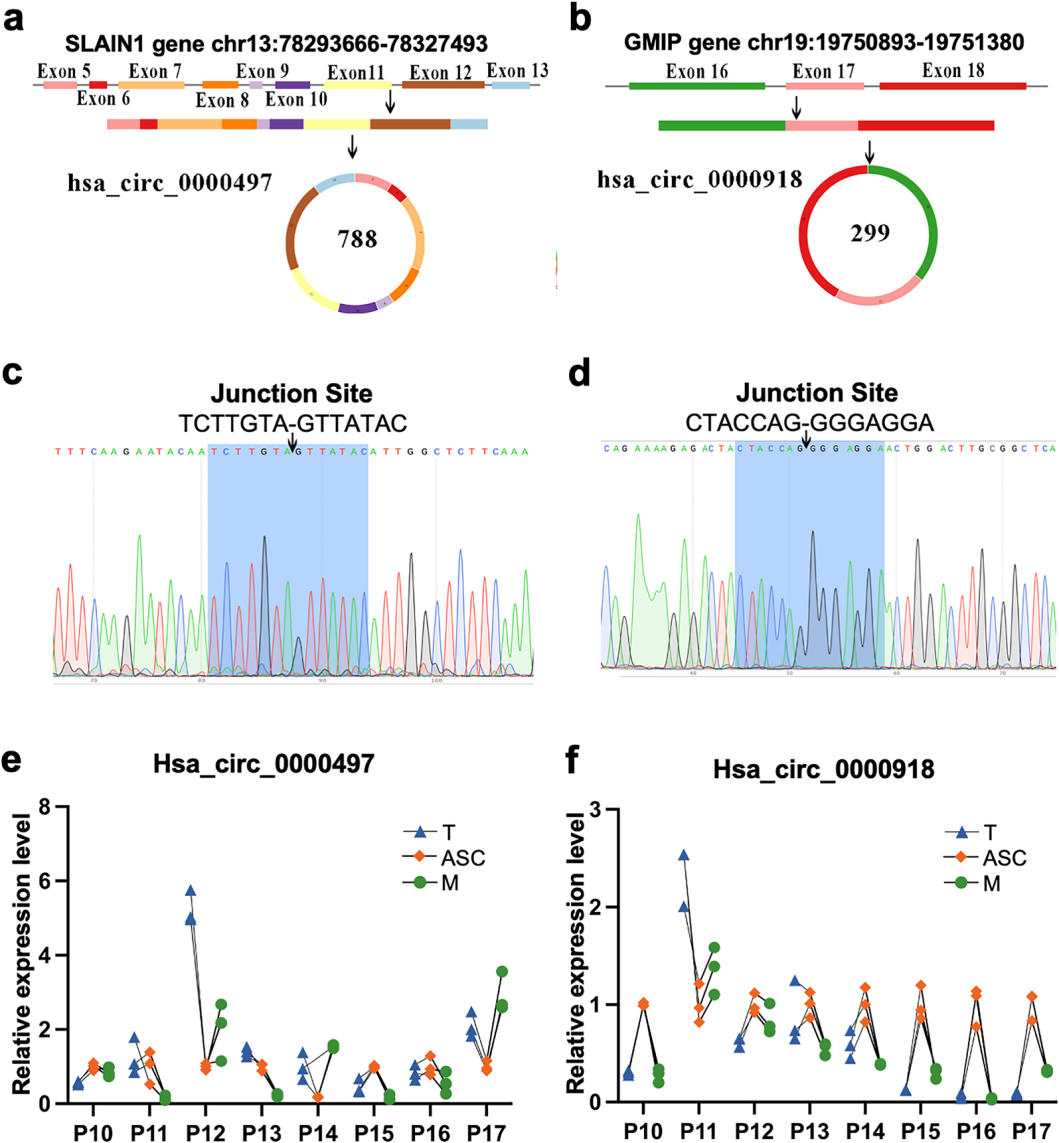
Validation of hsa_circ_0000497 and hsa_circ_0000918 differentially expressed in ASC. **a** Generation of hsa_circ_0000497 is produced 5–13 exons of its host genes SLAIN1 and is located at chr13:78,293,666–78,327,493. **b** Generation of hsa_circ_0000918 is produced 16–18 exons of its host genes GMIP and is located at chr19:19,750,893–19,751,380. **c** Back_splicing site in the qRT-PCR product of hsa_circ_0000497 by sanger sequencing. **d** Back_splicing site in the qRT-PCR product of hsa_circ_0000918 by sanger sequencing. **e** qRT-PCR analysis of the expression of circ_0000497 in 8 sets of paired samples, including primary tumor lesions (T), tumor cells from ascites (ASC) and metastatic lesions (M) in EOC patients. The expression of hsa_circ_0000497 was similar in T, ASC, and M. **f** qRT-PCR analysis of the expression of circ_0000918 in 8 sets of paired samples, including T, ASC and M in EOC patients. The expression level of hsa_circ_0000918 was significantly elevated in ASC than in T and M

The basic structural patterns of Hsa_circ_0000497 and hsa_circ_0000918 were firstly analyzed using a comprehensive cancer-specific circRNA database (CSCD). Hsa_circ_0000497 consists of 5–13 exons of its host genes SLAIN1 with the length of 788 nucleotides (Nt) and locates at chr13:78,293,666–78,327,493 (Fig. 4a). And Hsa_circ_0000918 consists of 16–18 exons of its host genes GMIP with the length of 299 Nts and located at chr19:19,750,893–19,751,380 (Fig. 4b). Both circRNAs were confirmed by the following Sanger sequencing using specific primers targeting back-splicing sites, respectively. They were closed-loop RNAs derived from reverse splicing of two exons (Fig. 4c, d). The expression of hsa_circ_0000497 and hsa_circ_0000918 were verified in 8 sets of paired samples, including T, ASC, and M. The expression of hsa_circ_0000497 was similar in T, ASC, and M (Fig. 4e), while the expression level of hsa_circ_0000918 was significantly elevated in ASC than in T and M (Fig. 4f).

### Hsa_circ_0000497 and hsa_circ_0000918 promoted migration and invasion of EOC cells via EMT in vitro

**Fig. 5:**
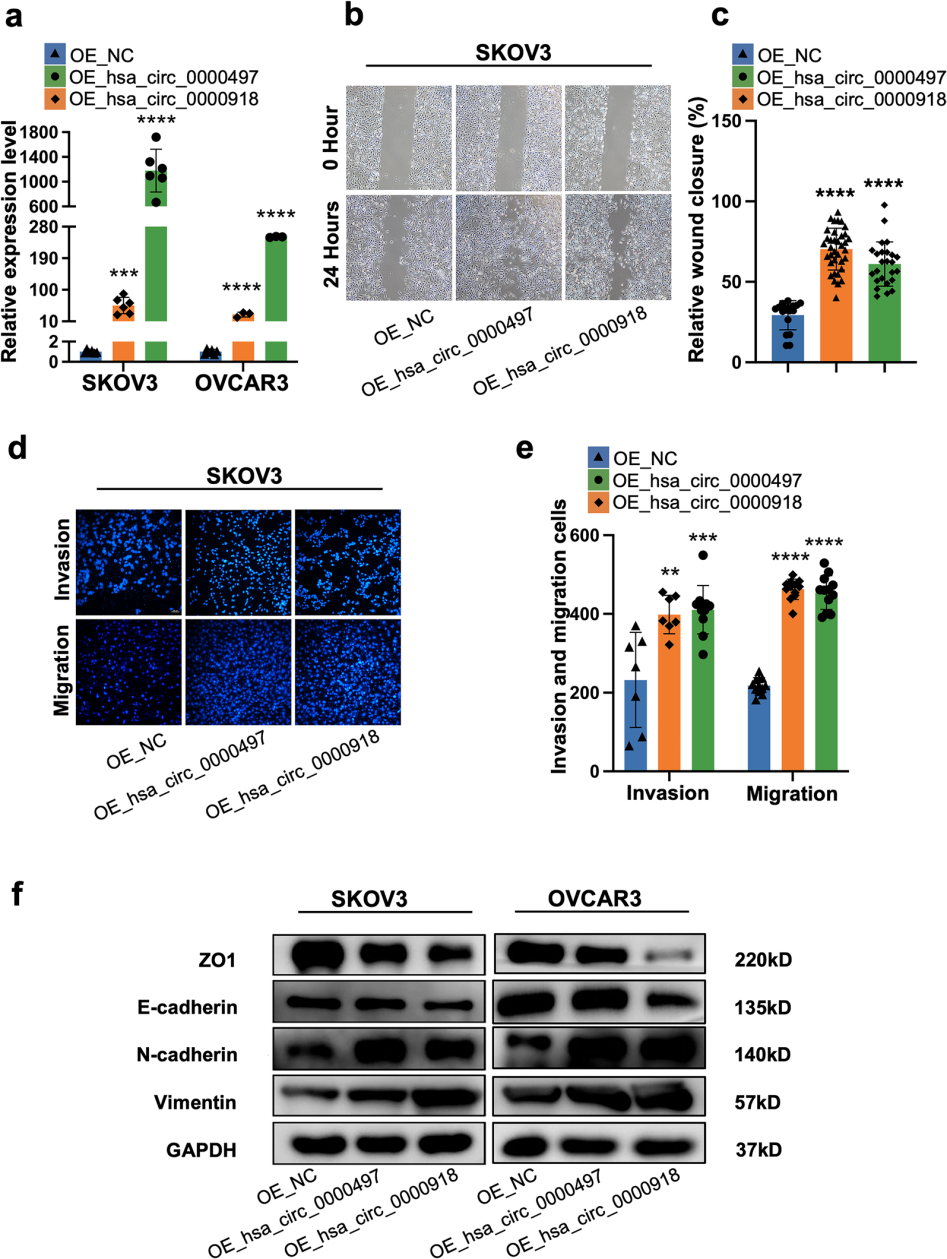
Over-expressed hsa_circ_0000497 and hsa_circ_0000918 promote the invasion and migration of ovarian cancer cell cells. **a** Expression of hsa_circ_0000497 and hsa_circ_0000918 in SKOV3 and OVCAR3 cells transfected with overexpressing plasmids. **b**, **c** Wound healing assay illustrated migrative ability after the overexpression of hsa_circ_0000497 and hsa_circ_0000918 in SKOV3 cells. **d, e** Transwell assay for migration and invasion was carried out to detect the invasive ability and migrative ability of skov3 cells after the overexpression of hsa_circ_0000497 and hsa_circ_0000918. **f** Western blot assay was used to detect the relative expression of E-cadherin, vimentin, N-cadherin, ZO-1, and GAPDH after the silencing of hsa_circ_0000497 and hsa_circ_0000918 in SKOV3 and OVCAR3 cellsb.

**Fig. 6:**
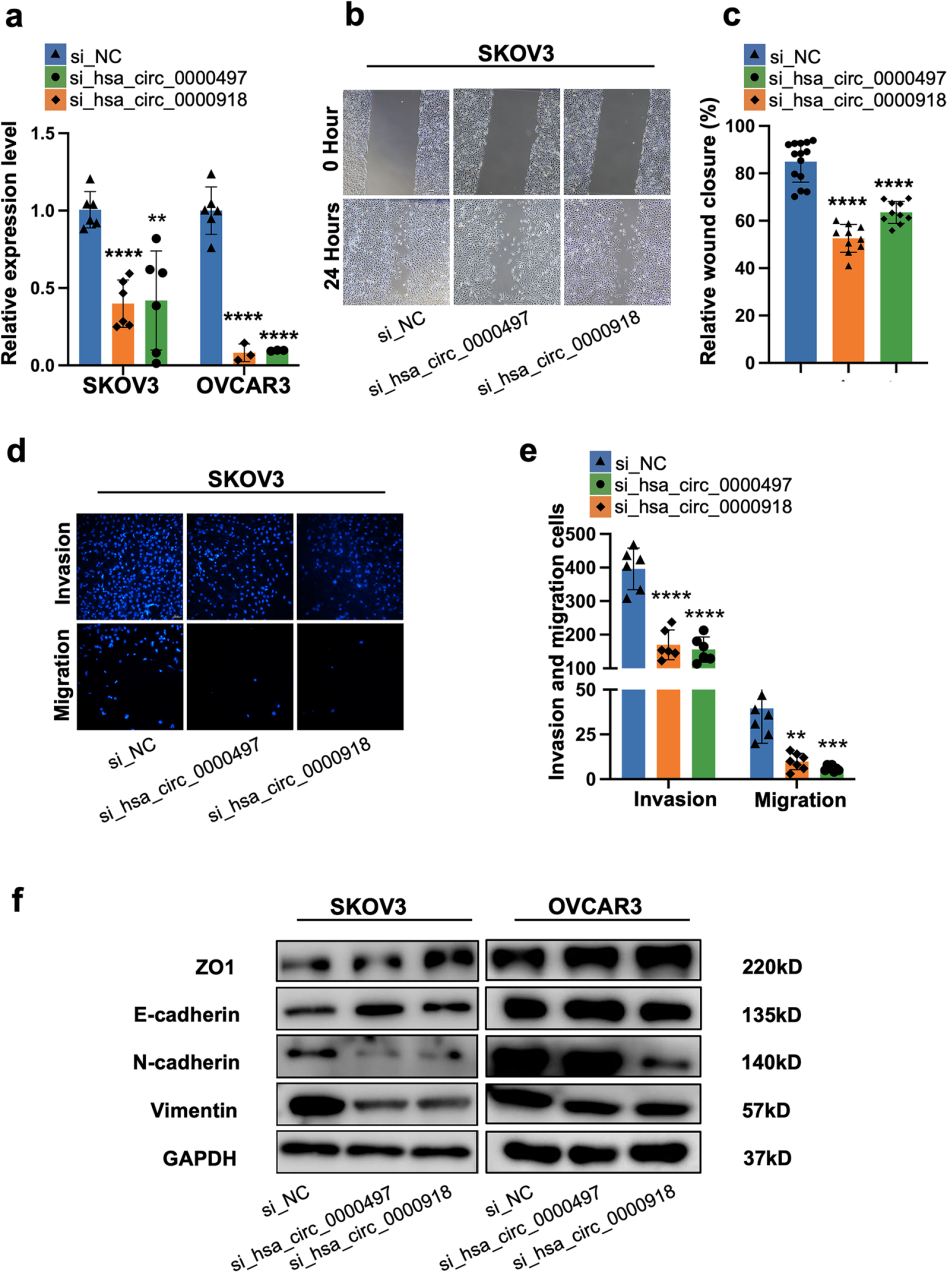
Silencing hsa_circ_0000497 and hsa_circ_0000918 inhibit the cell invasion and migration ovarian cancer cells. **a** Expression of hsa_circ_0000497 and hsa_circ_0000918 in SKOV3 and OVCAR3 cells transfected with siRNAs. **b**, **c** Wound healing assay illustrated migrative ability after the silencing of hsa_circ_0000497 and hsa_circ_0000918. **d,** **e **Transwell assay for migration and invasion was carried out to detect the invasive ability and migrative ability of skov3 cells after the silencing of hsa_circ_0000497 and hsa_circ_0000918. **f** Western blot assay was used to detect the relative expression of E-cadherin, vimentin, N-cadherin, ZO-1, and GAPDH after the silencing of hsa_circ_0000497 and hsa_circ_0000918 in SKOV3 and OVCAR3 cells

To verify the role of hsa_circ_0000497 and hsa_circ_0000918 in invasion and metastasis of EOC, the plasmids expressing circRNAs were transfected into human ovarian cancer cell lines (SKOV3 or OVCAR3), respectively. After validation of the overexpression (Fig. 5a), tumor cells were sent for wound healing assay. It showed that tumor cells with hsa_circ_0000918 or hsa_circ_0000497 overexpression all displayed enhanced migration ability (Fig. 5b, c). The following transwell assay also showed that overexpression of hsa_circ_0000918 or hsa_circ_0000497 enhanced cancer cell migration and invasion capabilities (Fig. 5d, e). In accordance with these assays, the protein expression of ZO1 and E-cadherin were down-regulated by circRNAs overexpression while the expression of N-cadherin and vimentin were up-regulated (Fig. 5f). These data together showed that both hsa_circ_0000497 and hsa_circ_0000918 promoted migration and invasion of tumor cells potentially via EMT processes. Tumor cells with down-regulated circRNAs were sent for metastasis analysis for further verification. Expression of circRNAs was found significantly down-regulated in tumor cells transfected with targeting siRNA, respectively (Fig. 6a). The wound-healing assay showed that down-regulation of hsa_circ_0000497 or hsa_circ_0000918 inhibited the migration of ovarian cancer cells (Fig. 6b, c). And the inhibition of migration and invasion by down-regulation of circRNAs was also observed by Transwell assay analysis (Fig. 6d, e, and Additional file 1: Fig. S2a, b). WB analysis found up-regulation of ZO1 and E-cadherin and down-regulation of N-cadherin and vimentin in tumor cells with downregulated circRNAs (Fig. 6f). These results suggested that both hsa_circ_0000497 and hsa_circ_0000918 circRNA promoted the migration and invasion of ovarian cancer via EMT processes in vitro.

### Construction of circRNA–miRNA–mRNA regulatory network

**Fig. 7:**
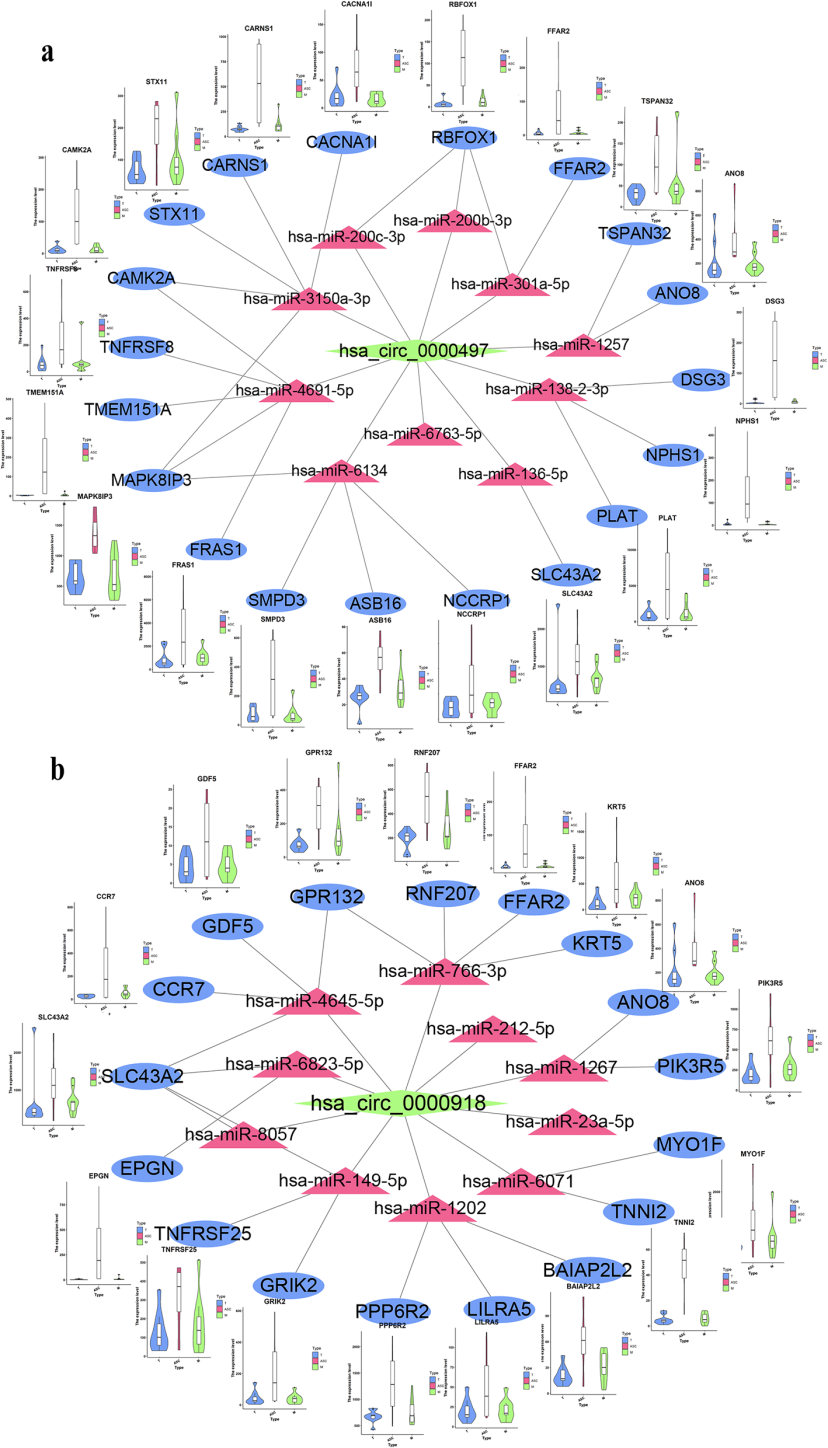
Construction of circRNA–miRNA–mRNA regulatory network in ascitic metastasis of ovarian cancer. **a** hsa_circ_0000497 co-expression network was constructed by Cytoscape software based on conjoint analysis of circRNA expression results. Red nodes represent circRNAs, green nodes represent miRNAs, and blue nodes represent mRNAs. **b** hsa_circ_0000918 co-expression network. Pink nodes represent circRNAs, cyan nodes represent miRNAs, and medium purple nodes represent mRNAs

Having found the promotional role of hsa_circ_0000497 and hsa_circ_0000918 in tumor cells, we intended to investigate the molecular mechanism underpinning via regulatory network construction. It had been widely reported that circRNA regulated downstream genes via sponge miRNAs [39]. So, we collected the potential targeting miRNAs of hsa_circ_0000497 and hsa_circ_0000918 from circBase Database (http://www.circbase.org/). 85 and 23 miRNAs were found to potentially bind with hsa_circ_0000497 and hsa_circ_0000918, respectively (Additional file 1: Table S4). And top 10 miRNAs according to the amount of miRNA binding sites of the circRNA in each set were selected for further analysis. Then miRDB (http://mirdb.org/) and TargetScan (http://www.targetscan.org/vert_72/) were used to identify targeting genes of candidate miRNAs. Total of 5875 mRNAs for hsa_circ_0000497 targeting miRNAs and 4897 mRNAs for hsa_circ_0000918 targeting miRNAs were obtained from both databases. Among these mRNAs, 36 genes were found differentially expressed in ASC based on our transcriptomic analysis compared with T and M (Additional file 1: Fig. S3), suggesting their potential correlation with hsa_circ_0000497 and hsa_circ_0000497. Based on these interaction data, we constructed circRNA–miRNA–mRNA networks for hsa_circ_0000497 and hsa_circ_0000918, respectively. As shown in (Fig. 7a), 8 miRNAs (hsa-miR-1257, hsa-miR-138-2-3p, hsa-miR-200b-3p, hsa-miR-200c-3p, hsa-miR-301a -5p, hsa-miR-3150a-3p, hsa-miR-4691-5p, and hsa-miR-6134) and 19 mRNAs (ANO8, DSG3, NPHS1, PLAT, RBFOX1, FFAR2, ASB16, CAMK2A, MAPK8IP3, CACNA1I, CARNS1, SLC43A2, FRAS1, TMEM151A, TNFRSF8, STX11, SMPD3, NCCRP1, and TSPAN32) were included in hsa_circ_0000497 ceRNA network. 7 miRNAs (hsa-miR-1202, hsa-miR-1267, hsa-miR-149-5p, hsa-miR-4645-5p, hsa-miR-6071, hsa-miR-6823-5p, and hsa-miR-766-3p) and 17mRNAs (BAIAP2L2, LILRA5, PPP6R2, ANO8, GRIK2, TNFRSF25, CCR7, TNNI2, EPGN, RNF207, KRT5, FFAR2, GDF5, GPR132, MYO1F, PIK3R5, and SLC43A2) were included in hsa_circ_0000918 ceRNA network (Fig. 7b). It showed that almost all mRNAs included in the network displayed higher expression levels in ASC than in T and M, and similar expression levels between T and M (Fig. 7, violin diagram). And expression patterns of these mRNAs were the same as that of hsa_circ_0000497 and hsa_circ_0000918, further suggesting their potential regulatory relationships in ovarian tumor cells. The miRNAs and mRNAs in the network may be the downstream targets through which hsa_circ_0000497 or hsa_circ_0000918 promoted metastasis of EOC.

## Discussion

In the present study, we investigated gene expression profiles including circRNA and mRNA of T, M, and ASC and identified different gene expression profiles among these tumors. Further analysis showed that ASC displayed mesenchymal phenotype while T and M had epithelial phenotype at the transcriptomic level. In addition, we identified core circRNAs correlated with mesenchymal phenotype and epithelial phenotype of ovarian tumors. The functional analysis found that hsa_circ_0000497 and hsa_circ_0000918, which up-regulated in ASC, promoted invasion and migration of tumor cells in vitro. Moreover, we found several miRNAs and mRNAs that were potentially involved in tumor cell metastasis regulated by hsa_circ_0000497 and hsa_circ_0000918. These findings uncovered the functional role of hsa_circ_0000497 and hsa_circ_0000918 in EOC metastasis, suggesting these circRNAs to be used as potentially therapeutic and diagnosis targets in EOC.

Peritoneal metastasis was observed in most EOC patients and was identified as one of the unfavorable prognosis factors of EOC [40]. Increasing evidence showed that ascites plays a crucial role in the peritoneal metastasis of EOC. It had been reported that multicellular aggregates (MCAs) or spheroids in ascites contributed to peritoneal metastasis [13, 41]. The theory of transcoelomic metastasis hypothesized that tumor cells detached from the primary tumor into the abdominal cavity accumulate and survive in the ascites, and then form malignant colonies when getting to a suitable location and epithelial-mesenchymal transition (EMT) of tumor cells played pivotal roles during the processs [40]. Ascites, as an ideal microenvironment of metastatic tumors, provides suitable conditions for tumor expansion, migration, and cell proliferation [10]. In the present study, we observed the mesenchymal phenotype of ASC and the epithelial phenotype of T and M via transcriptomic analysis (Fig. 2). The finding confirmed that peritoneal metastasis of EOC derived from primary tumor cells via EMT.

In addition, we identified circRNAs specifically up-regulated in ASC, which were involved in ascitic metastasis of ovarian cancer. We found that hsa_circ_0000497 and hsa_circ_0000918 promoted the invasion and metastasis of ovarian cancer in vitro and induced aberrant expression of EMT markers in ovarian cancer cells (Figs. 5, 6). circRNAs have been reported to play an important role in EMT and metastasis of cancer cells [42, 43]. Several studies have demonstrated that CircFGFR3 [23], hsa_circRNA_100395 [24], hsa_circ_0025033 [25], and hsa_circ_0005585 [26] promote ovarian cancer metastasis via EMT progression. Aberrantly expressed circRNAs among tissues of primary site, peritoneum, and lymph node metastasis from EOC patients further indicated the involvement of circRNAs in EOC metastasis [44]. These circRNAs were mostly found aberrantly expressed in primary or metastasis tissues, and few have been identified from ASC. Our findings illustrated that hsa_circ_0000497 and hsa_circ_0000918, which are enriched in ASC, were correlated with the mesenchymal phenotype of ASC, mediating E-M transition of EOC metastasis. Moreover, we also found that both circRNAs had roles in promoting the proliferation of tumor cells (Additional file 1: Fig. S4), suggesting their multiple roles in tumors. To our knowledge, both hsa_circ_0000497 and hsa_circ_0000918 have not been reported elsewhere until now. They should be novel therapeutic targets or prognosis biomarkers of EOC metastasis.

Serval mechanisms underlying the regulatory function of circRNAs had been reported previously [39]. CircRNAs were widely reported to function as competing endogenous RNAs (ceRNA). The ceRNA regulatory network plays a crucial role in the tumorigenesis and development of tumors including ovarian cancer. We constructed a circRNA-miRNA-mRNA regulatory network to explore the underlying molecular mechanisms of hsa_circ_0000497 and hsa_circ_0000918. Total of 15 miRNAs and 36 mRNAs were included in the ceRNA networks of hsa_circ_0000497 and hsa_circ_0000918. Many of them had been reported to be involved in cancer metastasis. For example, CCR7 and its ligand chemokine ligand 21 (CCL21) promote the EMT and up-regulate the stemness of oral squamous cell carcinoma by activating the JAK2/STAT3 signaling pathway [45]. PIK3R5 [46] and PLAT [47] positively regulated cancer metastasis and EMT of osteosarcoma cells and melanoma, respectively, while little is known about their roles in EOC metastasis. Since the results are based on computational biology, more thorough investigations are needed to verify which miRNAs/mRNAs are indeed involved in metastatic of EOC under hsa_circ_0000918 or hsa_circ_0000497 regulation. CricRNAs can also function via their host genes. SLAIN1, the host gene of hsa_circ_0000497, was characterized as embryonic stem cells of humans and mouse and expressed in the embryo, especially in the nervous system [48, 49]. GMIP, the host gene of hsa_circ_0000918, is a RhoA-specific GTPase-activating protein that regulates neuronal migration [50]. Hsa_circ_0000497 and hsa_circ_0000918 may regulate ovarian cancer metastasis via their host genes, although neither SLAIN1 nor GMIP is reported in tumor metastasis. In addition, we found both hsa_circ_0000497 and hsa_circ_0000918 contain open reading frames (ORFs, Additional file 1: Table S5) using ORF finder (https://www.ncbi.nlm.nih.gov/orffinder/). So they may also function via encoding proteins since circRNAs containing initial AUG followed by ORF can directly encode polypeptides [18]. However, all of these possibilities need more experiments for further verification.

We found up-regulation of hsa_circ_0000497 and hsa_circ_0000918 in ASC and identified their role in the promotion of EOC metastasis. There are still some limitations to our study. First, the sample size should be further expanded to verify the expression of hsa_circ_0000497 and hsa_circ_0000918 in ascites, primary and metastatic lesions of ovarian cancer. Second, only tumor cell lines were included for function analysis. Whether these circRNAs promote metastasis in vivo remains unclear. Xenograft mice models including patient-derived tumor xenograft (PDX), will be applied for further research in vivo in our future study. As for the underlying mechanism, only a regulatory network was constructed based on transcriptomic data. Whether or not are these downstream genes regulated by circRNAs need to be assessed via gain or loss of function assays in vitro and in vivo models. In addition, the capability of these circRNAs used as a therapeutic target or prognostic biomarkers and so on also needs more investigation.

## Conclusion

In summary, we found two circRNAs, hsa_circ_0000497 and hsa_circ_0000918, specifically up-regulated in ASC from EOC patients. Overexpression of these circRNAs enhanced the metastatic capability of EOC tumor cells via EMT. We also constructed the downstream regulatory network of these circRNAs and found out the miRNAs and differently expressed mRNAs potentially contributed to the role of circRNAs in EOC metastasis. These findings indicated that hsa_circ_0000497 and hsa_circ_0000918 contribute to intraperitoneal metastasis of EOC via ascites by regulating EMT of tumor cells. And they should be used as novel therapeutic targets or prognostic biomarkers of EOC intraperitoneal metastasis.

## Supplementary Information

**Additional file 1:** **Table S1**. The sequences of primers and siRNAs used for experiments in this study. **Table S2**. Antibodies used in this study. **Table S3**. Detailed information for EMT related gene sets. **Table S4**. Predicted miRNA of candidate circRNAs. **Table S5**. ORF of candidate circRNAs. **Figure S1**. CircRNAs differentially expressed in ascitic metastasis of ovarian cancer. **Figure S2**. Silencing hsa_circ_0000497 and hsa_circ_0000918 inhibit the cell invasion and migration of OVCAR3 cells. **Figure S3**. mRNAs differentially expressed in ascitic metastasis of ovarian cancer. **Figure S4**. over-expressing or silencing hsa_circ_0000497 and hsa_circ_0000918 promote or inhibit the cell proliferation ovarian cancer cells

## Data Availability

The datasets generated during and/or analyzed during the current study are available from the corresponding author on reasonable request.

## Abbreviations

EOC: Epithelial Ovarian Cancer
EMT: Epithelial-Mesenchymal Transition
circRNA: Circular RNA
PCA: Principal Component Analysis
ssGSEA: Single sample Gene Set Enrichment Analysis
DECs: Differentially Expressed CircRNAs
DEGs: Differentially Expressed Genes
T: Primary tumor lesions
ASC: Ascites tumor cells
M: Metastatic lesions
