# Supplementary material for: Hsa_circ_0000497 and hsa_circ_0000918 contributed to peritoneal metastasis of ovarian cancer via ascites
Source: J Transl Med. 2022 May 10;20:201. doi: 10.1186/s12967-022-03404-9 (PMC9092689; doi:10.1186/s12967-022-03404-9)
Supplement: Supplementary file 1 — Additional file 1: Table S1. The sequences of primers and siRNAs used for experiments in this study. Table S2. Antibodies used in this study. Table S3. Detailed information for EMT related gene sets. Table S4. Predicted miRNA of candidate circRNAs. Table S5. ORF of candidate circRNAs. Figure S1. CircRNAs differentially expressed in ascitic metastasis of ovarian cancer. Figure S2. Silencing hsa_circ_0000497 and hsa_circ_0000918 inhibit the cell invasion and migration of OVCAR3 cells. Figure S3. mRNAs differentially expressed in ascitic metastasis of ovarian cancer. Figure S4. over-expressing or silencing hsa_circ_0000497 and hsa_circ_0000918 promote or inhibit the cell proliferation ovarian cancer cells [file 12967_2022_3404_MOESM1_ESM.docx]

**Hsa_circ_0000497 and hsa_circ_0000918 contributed to peritoneal metastasis of ovarian cancer via ascites**

**Authors` names**: Ning Luo^1,2^, Zubaidan Sulaiman^1,2^, Chunyan Wang^1,2^, Jinye Ding^1,2^, Yingying Chen^1,2^, Biting Liu^1,2^, Zhongping Cheng^1,2*^, Shupeng Liu^1,2,3*^

**Authors` affiliations:** ^1^Department of Obstetrics and Gynecology, Tenth People’s Hospital, school of medicine, Tongji University, Shanghai, 200072, China

^2^Institute of Gynecological Minimally Invasive Medicine, school of medicine, Tongji University, Shanghai, 200072, China

^3^Department of Obstetrics and Gynecology, Putuo People’s Hospital, Tongji University, Shanghai 200060, China

***Corresponding authors:**

Shupeng Liu, Department of Obstetrics and Gynecology, Tenth People’s Hospital of Tongji University, Shanghai, 200072, China

Email: [lshup@tongji.edu.cn](mailto:lshup@tongji.edu.cn)

Telephone: 13761604076

and

Zhongping Cheng, Department of Obstetrics and Gynecology, Tenth People’s Hospital of Tongji University, Shanghai, 200072, China

Email：[mdcheng18@tongji.edu.cn](mailto:mdcheng18@tongji.edu.cn)

Telephone: 13816686812

Ning Luo, Zubaidan Sulaiman, and Chunyan Wang contributed equally.

Additional file 1: Table S1 The sequences of primers and siRNAs used for experiments in this study

| **Name** | **Sequence** |
| --- | --- |
| Primer_hsa_circ_0000497-F | GGTCACCGTGCGAAATAG |
| Primer_hsa_circ_0000497-R | GCAAAGGAGTCAGGGATT |
| Primer_hsa_circ_0000918-F | GGCAAGGTGTCCATTCAA |
| Primer_hsa_circ_0000918-R | TGAGCCGCAAGTCCAGTT |
| Primer_GAPDH-F | GTCGGAGTCAACGGATTTGG |
| Primer_CAPDH-R | CGGTGCCATGGAATTTGCC |
| siRNA_hsa_circ_0000497-F | AUACAAUCUUGUAGUUAUATT |
| siRNA_hsa_circ_0000497-R | UAUAACUACAAGAUUGUAUTT |
| siRNA_hsa_circ_0000918-F | AGACUACUACCAGGGGAGGTT |
| siRNA_hsa_circ_0000918-R | CCUCCCCUGGUAGUAGUCUTT |
| siRNA_NC-F | UUCUCCGAACGUGUCACGUTT |
| siRNA_NC-R | ACGUGACACGUUCGGAGAATT |

Additional file 1: Table S2 Antibodies used in this study

| **Antigens** | **Manufacturers** | **Applications** |
| --- | --- | --- |
| E-Cadherin | #14472, Cell Signaling Technology, Beverly, RA, USA | 1:500 for WB |
| N-cadherin | #14215, Cell Signaling Technology, Beverly, MA, USA | 1:500 for WB |
| ZO-1 | #8193, Cell Signaling Technology, Beverly, RA, USA | 1:1000 for WB |
| GAPDH | #5174, Cell Signaling Technology, Beverly, MA, USA | 1:5000 for WB |
| Vimentin | #5741, Cell Signaling Technology, Beverly, RA, USA | 1:1000 for WB |
| HRP Goat Anti Mouse IgG(H+L) | #AS003, ABclonal,China | 1:5000 for WB |
| HRP Goat Anti Rabbit IgG(H+L) | #AS014, ABclonal,China | 1:5000 for WB |

Additional file 1: Table S3 Detailed information for EMT related gene sets

| **Gene set** | **gene symbol** |
| --- | --- |
| **Mesenchymal Genesymbol** | ACVR1, ALX1, AXIN2, BCL9L, BAMBI, BMP2, BMP4, BMP7, CTNNBI, COL1A1, P98082, ENG, FOXC1, GLPR2, HDAC2, EZH2, ISL1, IL1B, IL6, LEF1, LOXL2, MDK, SMAD2, SMAD3, SMAD4, GCNT2, NOTCH1, Q99784, ODON, CRB2, J AG1, Q9H4X1, MTOR, SERPINB3, SDCBP, TGFBR1, TGFBR2, TIAM1, Q9NQB0, TGFB1I1, TGFB3, TWIST1, WWTP1, ZNF703, SNAI1, VIM, CDH2, FOXC2, SNAI2, GSC, FN1, MMP2, MMP3, SDC1 |
| **Epithelial Genesymbol** | ADIPOR1, BMP5, DAB21P, DACT3, EFNA1, FOXA1, FOXA2, FUZ, HPN, LDLRAD4, MAD2L2, NKX2, NOG, OVOL2, PBLD, PPP2CA, PTEN, SDHAF2, SFRP1, SFRP2, SMAD7, RAP, TBX5, TRIM62, USF3, VASN, TJP1, KRT, COL4A1, MUC1, DSP |

Additional file 1: Table S4 Predicted miRNA of candidate circRNAs

| **circRNA** | **Predicted miRNA** |
| --- | --- |
| hsa_circ_0000497 | hsa-miR-3150a-3p,hsa-miR-4691-5p,hsa-miR-6134,hsa-miR-6763-5p,hsa-miR-1257,hsa-miR-136-5p,hsa-miR-138-2-3p,hsa-miR-200b-3p,hsa-miR-200c-3p,hsa-miR-301a-5p,hsa-miR-301b-5p,hsa-miR-429,hsa-miR-4531,hsa-miR-4653-3p,hsa-miR-4659a-3p,hsa-miR-4659b-3p,hsa-miR-4700-5p,hsa-miR-5585-5p,hsa-miR-620,hsa-miR-6881-3p,hsa-miR-8089,hsa-miR-877-3p,hsa-miR-1-5p,hsa-miR-1225-3p,hsa-miR-1238-5p,hsa-miR-1294,hsa-miR-133a-5p,hsa-miR-15b-3p,hsa-miR-204-5p,hsa-miR-211-5p,hsa-miR-2115-5p,hsa-miR-27b-5p,hsa-miR-3129-3p,hsa-miR-3135b,hsa-miR-3199,hsa-miR-3200-3p,hsa-miR-331-3p,hsa-miR-3613-5p,hsa-miR-3618,hsa-miR-3664-3p,hsa-miR-3682-5p,hsa-miR-369-3p,hsa-miR-3692-5p,hsa-miR-3929,hsa-miR-409-3p,hsa-miR-4259,hsa-miR-4268,hsa-miR-4276,hsa-miR-431-5p,hsa-miR-4324,hsa-miR-4433a-3p,hsa-miR-4478,hsa-miR-4490,hsa-miR-4494,hsa-miR-4646-5p,hsa-miR-4666a-5p,hsa-miR-4742-3p,hsa-miR-4745-5p,hsa-miR-4758-5p,hsa-miR-485-5p,hsa-miR-491-5p,hsa-miR-499b-3p,hsa-miR-5002-5p,hsa-miR-508-3p,hsa-miR-5094,hsa-miR-548au-3p,hsa-miR-548o-3p,hsa-miR-5581-3p,hsa-miR-5683,hsa-miR-5706,hsa-miR-603,hsa-miR-6515-3p,hsa-miR-6740-5p,hsa-miR-6750-5p,hsa-miR-6783-5p,hsa-miR-6827-5p,hsa-miR-6832-3p,hsa-miR-6864-3p,hsa-miR-6884-5p,hsa-miR-7-5p,hsa-miR-7160-5p,hsa-miR-7162-3p,hsa-miR-8052,hsa-miR-8081,hsa-miR-876-5p |
| hsa_circ_0000918 | hsa-miR-766-3p,hsa-miR-4645-5p,hsa-miR-6071,hsa-miR-6823-5p,hsa-miR-8057,hsa-miR-1202,hsa-miR-1267,hsa-miR-149-5p,hsa-miR-212-5p,hsa-miR-23a-5p,hsa-miR-23b-5p,hsa-miR-2467-3p,hsa-miR-30a-3p,hsa-miR-3158-5p,hsa-miR-3194-3p,hsa-miR-3200-3p,hsa-miR-3907,hsa-miR-4435,hsa-miR-4742-5p,hsa-miR-509-5p,hsa-miR-5580-5p,hsa-miR-6760-3p,hsa-miR-6772-5p |

Additional file 1: Table S5 ORF of candidate circRNAs

| **circRNA_id** | **Label** | **Strand** | **Frame** | **Start** | **Stop** | **Length(nt\|aa)** |
| --- | --- | --- | --- | --- | --- | --- |
| hsa_circ_0000497 | ORF1 | + | 2 | 98 | >943 | 846\|281 |
|  | ORF2 | + | 3 | 549 | 818 | 270\|89 |
|  | ORF3 | + | 3 | 372 | 512 | 141\|46 |
|  | ORF4 | - | 2 | 649 | 515 | 135\|44 |
|  | ORF5 | - | 3 | 855 | 763 | 93\|30 |
|  | ORF6 | - | 3 | 387 | 298 | 90\|29 |
| hsa_circ_0000918 | ORF1 | + | 1 | 61 | >384 | 324\|107 |
|  | ORF2 | - | 2 | 266 | >3 | 264\|87 |

Additional file 1: Fig. S1 CircRNAs differentially expressed in ascitic metastasis of ovarian cancer


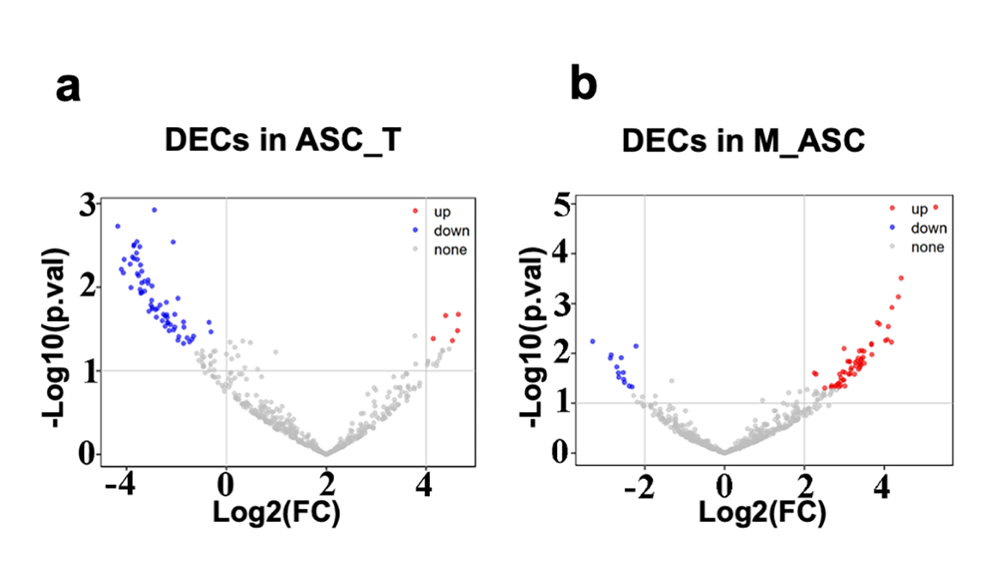


1. Volcano plot for DECs in ASC_T group. a total of 68 circRNAs (including 67 downregulated and 5 upregulated) identified by Foldchange>2 and p value<0.05.
2. Volcano plot for DECs in ASC_T group. a total of 71 circRNAs (including 14 downregulated and 57 upregulated) identified by Foldchange>2 and p value<0.05.

Additional file 1: Fig. S2 Silencing hsa_circ_0000497 and hsa_circ_0000918 inhibit the cell invasion and migration of OVCAR3 cells


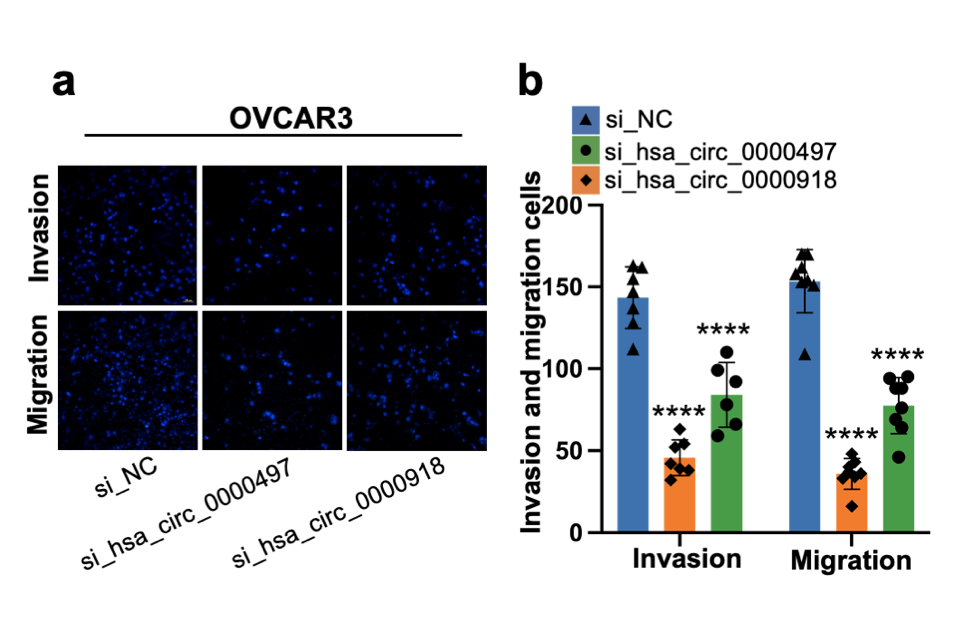


1. and b) Transwell assay for migration and invasion was carried out to detect the invasive ability and migrative ability of OVCAR3 cells after the silencing of hsa_circ_0000497 and hsa_circ_0000918

Additional file 1: Fig. S3 mRNAs differentially expressed in ascitic metastasis of ovarian cancer


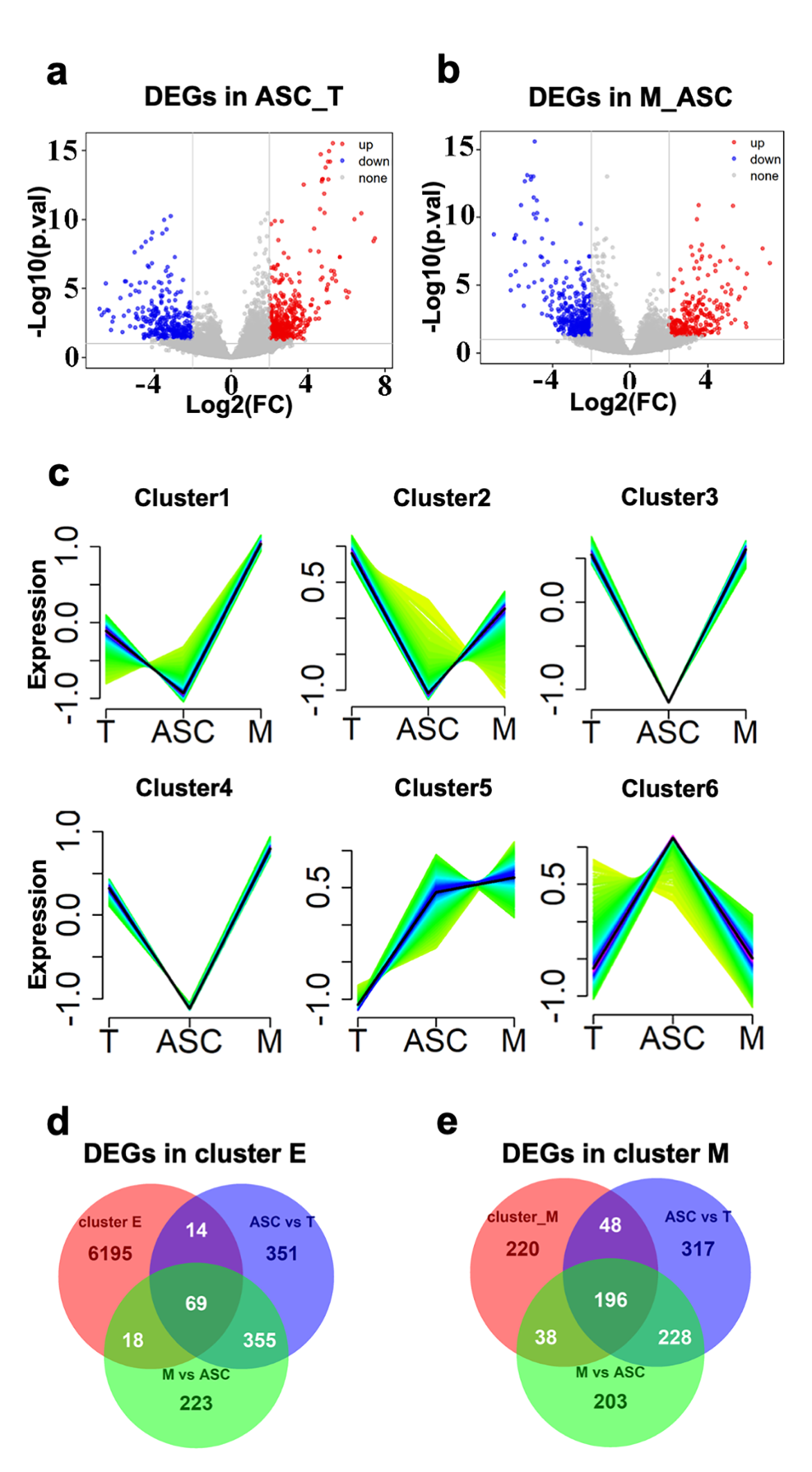


1. Volcano plot for DEGs in ASC_T group. a total of 1479 mRNAs (including 537 downregulated and 932 upregulated) in the T-ASC group identified by Foldchange>2 and adjusted p value<0.05.
2. Volcano plot for DEGs in ASC_T group. a total of 1350 mRNAs (including 908 downregulated and 442 upregulated) in the ASC- M group identified by Foldchange>2 and adjusted p value<0.05.
3. Clusters obtained by soft clustering analysis for mRNAs expression, Horizontal axis represents samples (T, ASC, M). The vertical axis represents expression changes. Cluster E (epithelial phenotype, including cluster1,3, and4) and cluster M (mesenchymal phenotype, including cluster6), respectively.
4. Venn diagram of overlapped mRNAs among the differentially expressed mRNAs between ASC and T groups, ASC and M groups, and cluster_E groups.
5. Venn diagram of overlapped mRNAs among the differentially expressed mRNAs between ASC and T groups, ASC and M groups, and cluster_M groups.

Additional file 1: Fig. S4 over-expressing or silencing hsa_circ_0000497 and hsa_circ_0000918 promote or inhibit the cell proliferation ovarian cancer cells


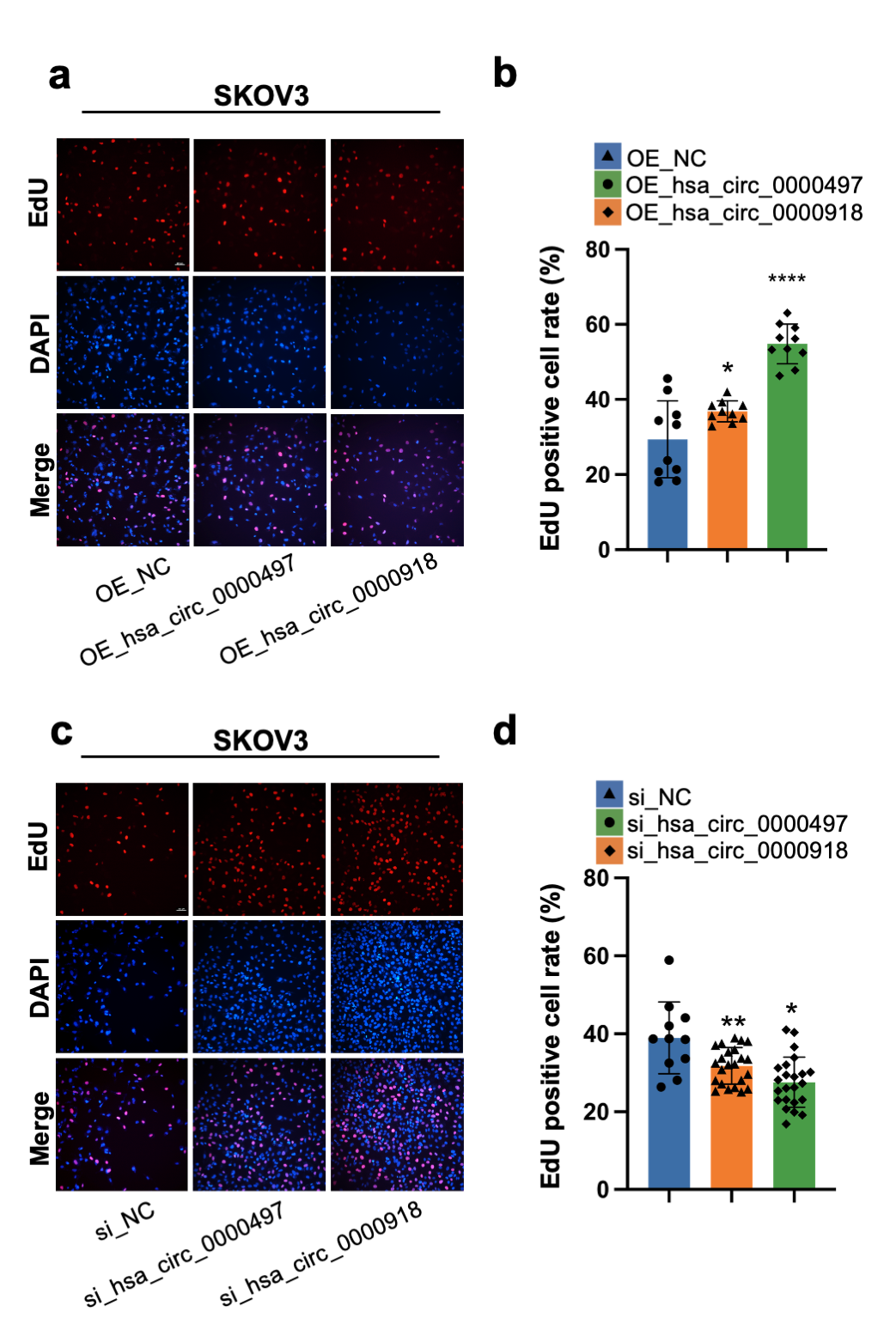


a) and b) EdU assay was used to detect cell proliferation of skov3 cells after the over-expressing of hsa_circ_0000497 and hsa_circ_0000918.

c) and d) EdU assay was used to detect cell proliferation of skov3 cells after the silencing of hsa_circ_0000497 and hsa_circ_0000918.
